# Influences of age-related positivity effect on characteristics of odor-evoked autobiographical memories in older Japanese adults

**DOI:** 10.3389/fpsyg.2022.1027519

**Published:** 2023-01-11

**Authors:** Kohsuke Yamamoto, Haruko Sugiyama

**Affiliations:** ^1^Faculty of International Studies, Osaka Sangyo University, Osaka, Japan; ^2^Sensory Science Research, Kao Corporation, Tokyo, Japan

**Keywords:** odor, autobiographical memory, aging, age-related positivity effect, olfaction

## Abstract

Older adults tend to remember past life events more positively than younger adults. This tendency is the *age-related positivity effect*. The present study examined whether this effect occurred for odor-evoked autobiographical memories. In total, 317 young and 181 older Japanese participants were asked to recall autobiographical events evoked by odors. Participants then completed the odor-evoked autobiographical memory questionnaire (OEAMQ) to measure the characteristics of the recalled memories. In the results, older participants recalled more positive memories than younger participants. Older participants also rated the OEAMQ subscales higher than the younger participants. Furthermore, there were significant positive correlations between the ratings of odor emotional characteristics and OEAMQ subscales. The age-related positivity effect was observed for odor-evoked autobiographical memories. The emotion aroused by odor played a significant role in some attributes of odor-evoked autobiographical memory. The age-related positivity effect in odor-evoked autobiographical memories has important implications for understanding the cognitive mechanisms of aging in autobiographical memory and olfaction and for applications in well-being and dementia in older adults.

## 1. Introduction

In 2015, the World Health Organization reported a dramatic increase in the proportion and absolute number of older people in the world’s population and a rapid increase in the rate of aging in many countries. These trends indicate a need for appropriate and rapid adaptation to an aging society (World Health Organization, 2015). Further, Japan has the world’s highest ratio of older people to the total population (Population Reference Bureau, 2022). Therefore, a critical need exists to support the well-being of older Japanese adults physically and psychologically.

In this study, we focused on the *age-related positivity effect* (ARPE), a trend that favors positive over negative stimuli in cognitive processing. Since the first explicit reference to the positivity effect in 2004 (Kennedy et al., 2004), more than 100 peer-reviewed articles have addressed the concept (for a review, see Reed and Carstensen, 2012). That is, relative to their younger counterparts, older people attend to and remember more positive than negative information (Gallo et al., 2011). In the context of *autobiographical memory*, or the memory of past personal events, ARPE refers to the tendency of older people to recall life events more positively than younger people do. When asked to recall autobiographical events in response to retrieval cues (e.g., words such as “friends” and “school”), the number of positive events tends to increase with age compared to adverse events (Dijkstra and Kaup, 2005; Ros and Latorre, 2010). Older adults also tend to rate retrieved events more positively than younger adults (Comblain et al., 2005). Schlagman et al. (2006) also conducted a content analysis of involuntary autobiographical memories recorded over a week in young and older adults and found that both groups recalled typical positive content memories to the same extent, whereas older adults recalled fewer typical negative content memories.

ARPE is a phenomenon based on the socio-emotional selectivity theory (Carstensen et al., 2006), which argues that perceptions of time frames play an important role in indicating motivational change (Mather and Carstensen, 2005; Charles and Carstensen, 2010). When the future is long and vague, knowledge acquisition and exploration help prepare individuals for the various uncertain challenges that lie ahead. Conversely, when the time horizon (i.e., limited remaining lifespan) is shorter, future-oriented goals related to long-term preparation become less important and present-oriented goals related to emotional meaning, emotional regulation, and well-being gain priority. Hence, in cognitive processing, older people pay more attention to and remember more positive information than negative information compared to younger people. As a result, ARPEs arise.

In this study, we investigate whether ARPE occurs in odor-induced autobiographical memory because two odor features suggest the possibility. First, previous studies have shown that odors are powerful cues for evoking autobiographical memories (Herz and Cupchik, 1992; Larsson and Willander, 2009; Larsson et al., 2014; Saive et al., 2014; Hackländer et al., 2019). Second, odor has a strong relationship with emotions and can efficiently induce them (Engen, 1982; Herz, 2002). For example, Herz and Cupchik (1992) reported that odor-evoked memories are highly emotional, vivid, specific, rare, and remote. Subsequent studies have confirmed these findings, indicating that memories associated with odors evoke a more powerful feeling of going back in time to the event’s occurrence compared to memories evoked by verbal labels and visual and auditory cues (Chu and Downes, 2002; Herz and Schooler, 2002; Herz, 2004; Willander and Larsson, 2006, 2007).

Human olfaction declines during normal aging in sensitivity and identification (Attems et al., 2015). However, the overall function does not necessarily decline. There are intriguing findings related to odor-induced autobiographical memory in older people. For example, autobiographical memories in older adults are more likely to be recalled when a word is accompanied by its corresponding odor than when it is presented alone (Maylor et al., 2002). Willander and Larsson (2006, 2007) reported that older people could evoke autobiographical memory through odor and that the evoked memories were from an earlier life era than those elicited by visual or verbal cues. Chu and Downes (2000) also reported comparable results. Even among older adults with Alzheimer’s disease, an odor might serve as a potent cue for autobiographical memory retrieval (El Haj et al., 2017; El Haj, 2021). These findings suggest that ARPE may be powerfully evoked *via* odor-induced autobiographical memory. However, this association or comparison between younger and older people remains under-investigated. Therefore, the first objective of this study was to examine whether ARPE occurs for odor-evoked autobiographical memories.

Additionally, we focused on the possibility that ARPE affects various memory characteristics. The relationship between emotion and memory has long been studied (e.g., Bower, 1981), and emotional state (e.g., mood) during recall has been argued to serve as a memory retrieval cue that activates emotional and related information in memory. Furthermore, previous research suggests that positive emotional information can create more specific retrieval cues than negative emotional information (Schaefer and Philippot, 2005; Schulkind and Woldorf, 2005; Berntsen and Rubin, 2006; Rasmussen and Berntsen, 2009). Schaefer and Philippot (2005) observed that positive autobiographical memories contain more details than negative or neutral memories. Talarico et al. (2004) also reported that autobiographical memories featuring high-intensity emotions have more details than those with low-intensity emotions. Conway (2005) suggested that autobiographical memories containing positive emotions or associations contain more subjective characteristics than negative or neutral memories. Thus, ARPE may enhance various memory characteristics. Luchetti and Sutin (2018) reported that older adults rate autobiographical memory characteristics (e.g., vividness) higher than younger adults. As mentioned earlier, odors increase the vividness and specificity of recalled autobiographical memories compared to other stimuli (Chu and Downes, 2002; Herz and Schooler, 2002; Herz, 2004). This phenomenon was confirmed in older adults (Willander and Larsson, 2006, 2007). However, no comparison has been made with younger adults. Accordingly, older people may recall more vivid and detailed memories than younger people due to ARPE.

These objectives were investigated using data from the odor-evoked autobiographical memory questionnaire (OEAMQ). Previous studies have developed various psychological measures of the multidimensional characteristics of autobiographical memory to elucidate its structure and cognitive mechanisms (e.g., Johnson et al., 1988). As mentioned earlier, the characteristics of autobiographical memory induced by odor stimuli have been studied in the past, but the measures have varied across studies, and no consistent measure has been developed. Against this background, Yamamoto and Sugiyama (2017) developed the OEAMQ by collecting various items assessing the characteristics of autobiographical memory, having participants evaluate memories recalled by odor cue and performing factor analysis on the results. The questionnaire has a seven-factor structure and adequate to good reliability (Cronbach’s alpha = 0.71〜0.95) has been confirmed. Additionally, previous studies (e.g., Ehrlichman and Halpern, 1988; Yamamoto and Nomura, 2010) have repeatedly found that pleasant and unpleasant emotional properties of odor cues influence various recalled memory characteristics. Yamamoto and Sugiyama attempted to examine the validity of this scale by showing the influence of emotional valence properties of odor cues on each factor of the OEAMQ with reference to these results. Their results showed that the affective valence rating of the odor cue was associated with the OEAMQ subscale scores, confirming that the questionnaire has adequate validity. Four of the seven factors (*retrospective recollection, clarity, time information, and perceptual experience*) were similarly identified in a previous study that used a Japanese version of the Memory Characteristics Questionnaire (Takahashi and Shimizu, 2007). The three other factors are new and specific for olfactory memory: *future action, emotion, and nostalgia*. The questionnaire was translated into English and back-translated by the author and experts from a translation corporation (see Table 1). The scale included items for an emotional valence rating (*positive–negative*) to test the positivity effect. In relation to our first objective, if ARPE occurs, older adults will recall more positive memories than younger adults will. Furthermore, scores on some factors on the OEAMQ scale will be higher in older adults than younger ones for the second objective.

**Table 1:** Structure of the OEAMQ (odor-evoked autobiographical memory questionnaire) composite variables.

Factors and items	1	7
F1 emotion		
The impression of the odor in my memory of this event is	Very unpleasant	Very pleasant
The overall impression of the memory is	Very unpleasant	Very pleasant
If the object of the odor (e.g., things, person, and place) involved in the event is there, I want to touch it many times	Not at all	Strongly agree
F2 retrospective recollection		
The memory of the event has taught me	Little or nothing	A lot of things
I learned something important from the event	Not at all	Strongly agree
At the time, the event seemed like it would have serious implications for me	Not at all	Definitely
F3 clarity		
My memory of this event is	Vague	Very vivid
The accuracy of my memory of this event is	Very dubious	Not dubious at all
The positional relationship of objects in my memory of the event is	Vague	Clear
F4 time information		
My memory of the time when the event took place is	Vague	Clear
My memory of the year when the event took place is	Vague	Clear
My memory of the season when the event took place is	Vague	Clear
F5 future action		
As the event was recalled, some plans to be done have come to mind	Not at all	Definitely
As the event was recalled, something that I want to put into action has come to mind	Not at all	Very strongly
As the event was recalled, some new ideas or thoughts have come to mind	Not at all	Definitely
F6 perceptual experiences		
My memory of the event involves a smell	Little or none	A lot
My memory of the event involves a taste	Little or none	A lot
My memory of the event involves visual details	Little or none	A lot
F7 nostalgia		
As I recall the event, I feel that I had traveled back to the time when it happened	Not at all	Strongly agree
I want to go back to the time when the event happened	Not at all	Strongly agree
As I recall the event, I feel	A little or not at all nostalgic	Very nostalgic

Although the OEAMQ scales have been found to have adequate reliability and validity (Yamamoto and Sugiyama, 2017), the research has thus far only involved younger people. Therefore, this study evaluates reliability using Cronbach’s alpha coefficients for younger and older adults. In addition, as in Yamamoto and Sugiyama (2017), the emotional valence and arousal of the odor cues were also measured. Their correlations with the OEAMQ subscales were used to confirm scale validity.

## 2. Materials and methods

### 2.1. Participants

The initially recruited participants were 319 younger (159 female participants, *M* = 34.90 years, *SD* = 8.04, age range 20–49 years) and 183 healthy older adults (92 female participants, *M* = 68.32 years, *SD* = 5.11, age range 60–79 years), screened and recruited from the database of a Japanese research firm. During the screening, they subjectively assured the researchers that they did not have colds, nasal rhinitis, or allergies and had no smell aversions. In addition, participants confirmed their intention to partake in this study by telephone after receiving a brief explanation of the research.

### 2.2. Stimuli

Eight odors (peach, jasmine, mint, orange, bar soap, vanilla, *hinoki* wood (*Chamaecyparis obtusa*), and rose) were used. These scents were reported to elicit past personal memories by Japanese participants in previous studies (Tanigawara et al., 1994; Yamamoto and Sugiyama, 2017). Mint was derived from natural spearmint essence. The other seven odors were artificially mixed. As in Yamamoto and Sugiyama (2017), the results were examined by several fragrance experts and diluted with triethyl citrate based on general human odor sensitivity. Scent-free white cellulose beads were saturated with the prepared odors and put in small high-density polyethylene bottles. Each bottle was labeled with a random letter to prevent participants from identifying its contents.

### 2.3. Procedure

This study occurred entirely in participants’ homes, based on the research methods of Yamamoto and Sugiyama (2017). Only one odor sample was evaluated per day to reduce participants’ load and the complexity of the process. First, participants received four of eight samples by mail. An e-mail with instructions and a questionnaire link was sent every assessment day. The participants were asked to smell one specified odor sample and complete the questionnaire on a personal computer or smartphone.

The participants were informed that the questionnaire was related to memories recalled when they smelled the sample odors. If they did not recall any memories, they only assessed odor characteristics and moved on to the next trial (they were excluded from the data analysis). If multiple memories were elicited, they focused on the first one recalled. First, they described how old they were when the recalled event happened and the memory’s content. They also selected one of 10 options for at what age the event occurred. Next, they answered 21 questions from the OEAMQ, using a seven-point scale to rate the following five items on the perceptual and cognitive aspects of odors: emotional arousal [*“How much emotion was aroused by smelling the odor?”* (1 = *not at all*, 7 = *very much*)], emotional valence [*“How positive or negative is that emotion?”* (1 = *very negative*, 7 = *very positive*)], odor intensity [*“How intense is the odor according to you?”* (1 = *not at all*, 7 = *very strong*)], and familiarity [*“How familiar does the odor feel?”* (1 = *not at all*, 7 = *very much*)]. Finally, they were also asked to identify what the odor was and to rate their confidence in the identification [*“How confident are you about naming the odor?”* (1 = *not at all*, 7 = *very much*)].

This procedure was repeated over four consecutive days, followed by a break of 3 days, and then the last four samples were sent and evaluated similarly. There were four patterns of sample presentation order, one of which was applied to each participant randomly. Twenty-one questions of the OEAMQ were programmed to be presented in random order each time. They were asked to control environmental conditions for each at-home rating session, such as maintaining quiet and good ventilation. After completing all the tasks, each participant received a 1,000-yen gift certificate.

## 3. Results and discussion

Four participants who could not complete the entire procedure were excluded. The final analysis included data for 317 younger and 181 older participants.

The total number of recalled memory cases was 1,718 (67.32%) in the younger group and 1,163 (79.44%) in the older group (lower percentages were converted as 100% if all participants in each group were able to recall a memory in response to the eight different stimuli). Table 2 shows the number of recollections, the most selected age group, and examples of memories for each odor. Older adults had significantly higher memory recall rates than younger adults (*x^2^* = 66.80, *p* < 0.01, *df* = 1). For every odor except mint, older participants’ recalled memory rates were significantly higher than those of younger people. The mean number of odor-evoked memories per person was 6.70 (*SD* = 1.69) in the younger group and 7.39 (*SD* = 1.32) in the older group. Not all participants could evoke autobiographical memories for each odor. Therefore, the rating data were averaged for each participant irrespective of the number of odors that evoked memories. A normality test of the data confirmed little normality. Thus, data were compared using a Wilcoxon rank sum test and Spearman’s correlation analysis as a non-parametric test. Analyses were performed with SPSS statistical software (Version 27).

**Table 2:** Number of recollections, most selected age group, and examples of memory for each odor.

	Young participants			Older participants		
	Number of recollections	Most selected age group	Example of recollection	Number of recollections	Most selected age group	Example of recollection
Orange	236	10–19 years	I remember the grapefruit-flavored gum I used to have a lot in elementary school. I used to buy it as a snack when I traveled. (Male, 42)	154	10–19 years	In my elementary school age. Eating tangerines with my family in winter. (Female, 60)
Jasmine	161	10–19 years	It smells like the bath salts used at my home until I was a college student. It reminded me of my life at home. (Male, 23)	123	10–19 years	Around age 10, I was building a hide-out with friends in the high grass in my neighborhood. I used to get excited thinking about how we would decorate it today. (Female, 70)
Bar soap	234	under 10 years	I remember making soap bubbles in the bath when I was a child. I was fascinated for dozens of minutes because it was so interesting to watch the bubbles softly fly. (Female, 21)	148	10–19 years	It reminds me of the feeling of relaxing in a hot spring on a trip when I was in high school. (Male, 65)
Vanilla	247	under 10 years	When I was in kindergarten, my mother bought me ice cream to eat on the way home. It smelled so sweet and good. (Male, 49)	169	10–19 years	Eating ice cream with family during summer vacation. I remember eating vanilla ice cream and looking forward to getting another one if “you have won!” was written on the stick. (Female, 64)
Peach	213	10–19 years	I remember a pen pal sent me scented letters in my junior high school days. When I opened them, I smelt a faint fragrance and thought, “how nice it is to put fragrance on a letter.” (Female, 34)	149	10–19 years	In the 4th–6th grade of elementary school. There was a plum tree at home, and every year I would pick delicious plums and eat them. (Female, 61)
Hinoki wood	186	10–19 years	A few years ago, when I went shopping with my mother, she was examining the bath salts in a drugstore, comparing prices, fragrances, and efficacies. (Male, 22)	137	10–19 years	Final year of elementary school. Being the only person to receive an award for effort at the graduation ceremony. (Male, 73)
Mint	264	10–19 years	My first kiss with my first boyfriend when I was in high school. (Female, 23)	153	10–19 years	It was in high school. I remembered the refreshing aroma of chewing and tasting mint gum with my friends. (Male, 65)
Rose	177	10–19 years	When I was studying overseas at a university in the United States, I lived in a women’s dormitory. I remember doing my laundry in the basement laundry room. (Female, 36)	130	10–19 years	When I was 12 years old. I remembered the soap sent to me by my aunt who married a man in Hawaii. (Male, 64)

### 3.1. Reliability and validity of the odor-evoked autobiographical memory questionnaire

Reliability coefficients were calculated for each factor for the OEAMQ scores of younger and older adults. The results showed that emotion α = 0.95, retrospective recollection α = 0.96, clarity α = 0.96, time information α = 0.94, future action α = 0.97, perceptual experience α = 0.84, and nostalgia α = 0.89 in the young, and emotion α = 0.97, retrospective recollection α = 0.98, clarity α = 0.98, time information α = 0.97, future action α = 0.98, perceptual experience α = 0.92, and nostalgia α = 0.95 in the old people.

Ratings of the cognitive characteristics of odor are presented in Table 3. A Wilcoxon rank sum was conducted on the mean ratings between the two groups to examine the generational differences in odor’s cognitive characteristics. Table 3 shows that the older group had significantly higher ratings on emotional valence and emotional arousal than the younger group. Familiarity was higher among the younger group than the older group. No difference in confidence was detected concerning identifications between the groups. The rating of odor intensity was lower in the older than younger adults but higher than the median score of 4, suggesting that the odor was perceived by all older participants.

**Table 3:** Mean score (SD) on odor cognitive characteristics by each group.

Ratings	Young	Older	Wilcoxon	*r*
Emotional valence	4.55 (0.82)	4.81 (0.88)	3.34^****^	0.15
Emotional arousal	3.66 (0.74)	4.17 (0.76)	7.23^****^	0.32
Odor intensity	5.10 (0.72)	4.89 (0.73)	2.78^***^	0.13
Familiarity	4.82 (0.86)	4.62 (0.83)	2.59^*^	0.11
Confidence of naming	3.99 (1.03)	3.88 (1.06)	0.51	0.02

^****^p < 0.001, ^***^p < 0.005, ^*^p < 0.05.

A Spearman correlation analysis was performed between scores on the OEAMQ subscales and the odor’s emotional characteristics (*valence* and *arousal*) for each group to examine scale validity. Table 4 shows that significant positive correlations between the OEAMQ factors and odor emotional characteristics ratings were confirmed for both groups. These results confirm adequate reliability and validity of the OEAMQ in older participants.

**Table 4:** Correlation coefficients between the OEAMQ subscales and odor-induced emotional items by each group.

	F1 EM	F2 RR	F3 Cl	F4 TI	F5 FA	F6 PE	F7 NO
Young people							
Emotional valence	0.83^**^	0.44^**^	0.41^**^	0.33^**^	0.32^**^	0.49^**^	0.60^**^
Emotional arousal	0.09	0.31^**^	0.24^**^	0.30^**^	0.34^**^	0.26^**^	0.21^**^
Older people							
Emotional valence	0.87^**^	0.62^**^	0.62^**^	0.57^**^	0.58^**^	0.68^**^	0.75^**^
Emotional arousal	0.47^**^	0.50^**^	0.41^**^	0.40^**^	0.52^**^	0.47^**^	0.49^**^

^**^p < 0.01, F1 EM, emotion; F2 RR, retrospective recollection; F3 CL, clarity; F4 TI, time information; F5 FA, future action; F6 PE, perceptual experiences; F7 NO, nostalgia.

### 3.2. Age-related positivity effects

The first hypothesis was that ARPE would occur for odor-evoked autobiographical memory. The valence rating score of the memories from the OEAMQ (“The overall impression of the memory is 1 = *very unpleasant*; 7 = *very pleasant*”) was used to examine this hypothesis. A Wilcoxon rank sum test revealed that the older group (*Mean* = 5.09, *SD* = 0.81) had significantly higher scores than the younger group [*Mean* = 4.70, *SD* = 0.73, *W* = 5.11, *p* < 0.001, *r* = 0.23]. Thus, the results showed that ARPE occurred for odor-evoked autobiographical memory. In addition, there are gender differences in the olfactory function (Sorokowski et al., 2019). The study preliminarily examined whether gender differences in ARPE could be identified. Analysis of the Wilcoxon rank sum test showed significant differences for both younger and older adults (male participants, *W* = 4.03, *p* < 0.001; female participants, *W* = 3.41, *p* < 0.001). Both younger and older adults indicated that female participants (younger, *Mean* = 4.79, *SD* = 0.72; older, *Mean* = 5.17, *SD* = 0.88) recalled positive events more than male participants (younger, *Mean* = 4.61, *SD* = 0.73; older, *Mean* = 5.00, *SD* = 0.73). Although a gender difference was observed, there may not be a direct relationship between the occurrence of ARPE and gender, since it was found in both age groups.

### 3.3. Memory characteristics

Ratings for the OEAMQ items were averaged for each factor (Table 5). A Wilcoxon rank sum test compared the means of each factor between the younger and the older groups to examine whether ARPE influenced memory characteristics. Table 5 shows that the older group had significantly higher ratings than the younger group for all OEAMQ factors. As expected, the results indicate that ARPE affected memory characteristics.

**Table 5:** Mean score (SD) on OEAMQ by each group.

Factor	Young	Older	*Wilcoxon*	*r*
F1 emotion	4.65 (0.71)	5.05 (0.81)	5.51^****^	0.25
F2 retrospective recollection	3.36 (1.06)	4.17 (1.08)	7.62^****^	0.34
F3 clarity	3.78 (1.08)	4.23 (1.09)	4.32^****^	0.19
F4 time information	3.30 (1.12)	3.85 (1.23)	4.85^****^	0.22
F5 future action	2.78 (1.09)	3.37 (1.14)	5.37^****^	0.24
F6 perceptual experiences	3.64 (0.91)	3.94 (0.95)	3.35^****^	0.24
F7 nostalgia	3.85 (1.04)	4.16 (1.06)	2.82^****^	0.13

^****^p < 0.001.

## 4. General discussion

We found that ARPE occurred *via* odor-evoked autobiographical memory. In addition, for both younger and older people, memory characteristics were influenced by the emotion evoked by odor. These results were consistent with both hypotheses. Although this study does not deal directly with well-being, it provides essential findings concerning the relationship between memory and positive emotions in older adults, valuable for bolstering declining physical and mental functions and improving their well-being. We will examine these findings from cognitive neuropsychology and cognitive psychology perspectives.

ARPE aroused by odor may be related to neuropsychological processes. For example, Herz et al. (2004) asked participants whether they could recall a positive memory with the sight and scent of a perfume. They were later presented with the odors and pictures of the recollected perfumes in the fMRI (functional magnetic resonance imaging) while retrieving these memories. The results showed that odor-evoked memories were related to more robust amygdala and hippocampal region activations than picture-cued recollections. Arshamian et al. (2013) demonstrated that along with the amygdala and hippocampus, odor-evoked autobiographical memories also activated the limbic and paralimbic cortices of the piriform cortex and entorhinal cortex and an extended limbic network (Morgane et al., 2005), including the parahippocampus, insular cortex, and the orbitofrontal cortex. According to Mather (2016), aging is a multifaceted process that involves the interaction of brain regions and neurotransmitter systems not uniformly affected by aging. It is associated with an apparent decline in physical and cognitive processes. Nevertheless, emotional functioning fares well. Consistent with this behavioral profile, two core emotional brain regions, the amygdala and ventromedial prefrontal cortex, may show minor structural and functional decline upon aging compared with other areas (Mather, 2016). This construct may support odor-aroused ARPE.

How should we interpret ARPE from a cognitive psychology perspective? According to the self-memory system (Conway and Pleydell-Pearce, 2000; Conway, 2005), knowledge of one’s past is distributed across a hierarchical system. The model distinguishes between two different autobiographical memory retrieval processes, generative and direct. In *generative retrieval*, cues entail an intentional cyclic and elaborative search process of information until a specific memory is formed, a process associated with verbal cues. In *direct retrieval*, recollection is automatic and effortless and immediately activates a representation of an event in memory. The retrieval process through odor cues depends on direct search (e.g., Conway and Pleydell-Pearce, 2000; Chu and Downes, 2002; Conway, 2005). Conway (2005) suggested that direct retrieval is based on the encoding specificity principle of Tulving and Thomson (1973). The general principle is that matching the encoding context of information during recall assists in episodic memory retrieval. It provides a framework for understanding how conditions present while encoding information relates to memory and recall of that information. In the case of autobiographical memory, the more specific the retrieval cue, the more detailed the memory of an event.

As mentioned earlier, previous research suggests that positive emotional information creates more specific retrieval cues than negative (Schaefer and Philippot, 2005; Schulkind and Woldorf, 2005; Berntsen and Rubin, 2006; Rasmussen and Berntsen, 2009), as it is essential for good mental health to maintain a positive self-concept. This study found correlations between the emotional and memory characteristics of odors. According to Engen (1982), odors are so powerfully connected to emotions that they function as a type of emotional judgment. Furthermore, they are so intensely emotion-inducing that they have been used as an effective means of eliciting moods in emotion research (Herz, 2002). Therefore, the emotional information evoked by an olfactory cue during retrieval matches the emotional characteristics of the event evoked during encoding, facilitating retrieval. Older adults rated odors’ emotional arousal and pleasantness more highly than younger adults, suggesting that emotional cues were more readily available to older adults than younger ones and that more direct retrievals occurred. Therefore, the older adults rated the various memory characteristics higher than the younger ones did. Thus, ARPE occurred.

It should be noted that a response bias could have resulted, as older people are more likely to select higher ratings for OEAMQ questions. However, in some instances, younger people rated odors higher than older people for cognitive characteristics of odors, and in other places, no differences were found between the groups. Hence, the probability of response bias by older adults is low.

Other causes of ARPE need further investigation. One of them is the amount of memory retained by disparate generations is different. Older people have lived longer than their younger counterparts and therefore have a greater range of varied memories. Additionally, the total number of positive memories in older people is higher than in younger ones. This implies that older people have a greater selection of positive memories than younger people at the time of memory recall. As, positive memories are more easily accessed than negative memories for mental health and self-maintenance (Conway, 2005), it is plausible that older people are more likely to recall positive memories than younger people. Accordingly, the timing of the occurrence of recalled autobiographical memories in older and younger adults was analyzed and summarized in the Supplementary Table S1. It demonstrated that events from the 10–19 age group were recalled more frequently in both groups. However, the older adults exhibited a certain amount of recall of events from other periods as well. Therefore, several factors can cause ARPE, and further research is needed.

However, several questions remain unanswered. First, the current study targeted only healthy young and older adults. As mentioned previously, for people with Alzheimer’s disease, prior studies investigated the subjective reliving of odor-evoked autobiographical memories (El Haj et al., 2017; Glachet and El Haj, 2019; El Haj, 2021). El Haj et al. (2017) examined whether odor-evoked autobiographical memory is an involuntary process that shares similarities with music-evoked autobiographical memory in those with mild Alzheimer’s disease. Participants with Alzheimer’s disease showed better specificity, emotional experience, mental time travel, and retrieval time after odor and music exposure than those in the control condition (without odor and music). Similar beneficial effects of odor and music exposure were observed for autobiographical characteristics, which improved more after odor than music exposure. Based on these results, ARPE could occur not only in healthy older adults but also in people with Alzheimer’s disease in the future. Future research should address this issue in developing dementia prevention programs.

Second, this study was conducted only among Japanese people, and the odors included were specific to Japan. Previous studies have shown that odor perception phenomena can be culturally differentiated (Ayabe-Kanamura et al., 1998). In addition, culture impacts autobiographical memory (Herz, 2004; Alea et al., 2015). Therefore, to examine the reliability of the results of this study further, experimental investigations should be conducted with participants from other cultures using a variety of odor stimuli.

Third, experimental control issues exist. Online experiments have been conducted in recent years, partly due to the COVID-2019. As a result, these studies may be limited in their validity (Semmelmann and Weigelt, 2017). For example, the present study collected data at the participants’ homes; however, we attempted to procedurally reduce their workload and control their motivation, assessment timing, and environmental conditions. Nevertheless, strict control was not guaranteed. Thus, future reliability studies should be performed as laboratory experiments or in more controlled experimental settings.

Fourth, positive odor stimuli were used in this study and negative odors were not used. Indeed, the odor stimuli were generally positively rated in the evaluation of the experiment. Therefore, it remains to be investigated whether negative odors also elicit ARPE, as Ehrlichman and Halpern (1988) suggest that negative odors evoke negative emotions and the associated negative memories. If this is the case, negative odors may not elicit ARPEs. Although recent ethical issues have made the use of negative odor stimuli difficult, there is a need for further investigation.

In the present study, as in others, self-reporting was used to screen healthy older adults without deficits in olfactory ability. However, future studies will need to measure and screen olfactory abilities in detail. Several studies have shown that decreased olfaction is quite common among older adults (Doty and Kamath, 2014; Attems et al., 2015). Previous studies have indicated that it presents in >50% of individuals aged between 65 and 80 years and 62–80% of those >80 years of age (Attems et al., 2015). In Japan, an olfactory identification test for older adults has been developed and validated (Gotow et al., 2021). Additionally, smell dysfunction significantly influences physical well-being, quality of life, nutritional status, and everyday safety and is associated with increased mortality (e.g., Pinto, 2011). However, this study’s results indicated the superiority of older adults in emotional aspects of odor-induced autobiographical memory. Emotions evoked by odor could play a vital role in promoting the well-being of older people. Therefore, further studies should be conducted more directly, focusing on cognitive mechanisms underlying ARPE and odor-induced autobiographical memory.

## Data availability statement

The raw data supporting the conclusions of this article will be made available by the authors, without undue reservation.

## Ethics statement

The studies involving human participants were reviewed and approved by Local Research Committee of Osaka Sangyo University. The patients/participants provided their written informed consent to participate in this study.

## Author contributions

KY and HS: conceptualization, data curation, funding acquisition, and investigation. KY: formal analysis, project administration, and writing – original draft. HS: writing – review and editing. Both authors contributed to the article and approved the submitted version.

## Funding

This study was supported by a Grant-in-Aid for Scientific Research (JSPS KAKENHI; Grant numbers 17 K13924 and 22 K03092) and the Kao Corporation.

## Conflict of interest

HS was employed by company Kao Corporation.

The remaining authors declare that the research was conducted in the absence of any commercial or financial relationships that could be construed as a potential conflict of interest.

The authors declare that the Kao Corporation funded this study, where HS was employed and involved in the study design, execution, and revision of the manuscript.

## Publisher’s note

All claims expressed in this article are solely those of the authors and do not necessarily represent those of their affiliated organizations, or those of the publisher, the editors and the reviewers. Any product that may be evaluated in this article, or claim that may be made by its manufacturer, is not guaranteed or endorsed by the publisher.

## Abbreviations

ARPE: Age-related positivity effect
OEAMQ: Odor-evoked autobiographical memory questionnaire
