# Supplementary material for: Influences of age-related positivity effect on characteristics of odor-evoked autobiographical memories in older Japanese adults
Source: Front Psychol. 2023 Jan 11;13:1027519. doi: 10.3389/fpsyg.2022.1027519 (PMC9875735; doi:10.3389/fpsyg.2022.1027519)
Supplement: Supplementary file 1 [file Table_1.DOCX]

Supplementary Material

Influences of age-related positivity effect on characteristics of odor-evoked autobiographical memories in older Japanese adults

Kohsuke Yamamoto^*^, Haruko Sugiyama

*** Correspondence:** Kohsuke Yamamoto
[kyamamoto@int.osaka-sandai.ac.jp](mailto:kyamamoto@int.osaka-sandai.ac.jp)

# Supplementary Table

**Supplementary Table 1.** **Classification of the timing of memory occurrence in each group**

|  | Younger | Older |
| --- | --- | --- |
| Under 10 years | 444(26%) | 113(10%) |
| 10–19 years | 529(31%) | 236(20%) |
| 20–29 years | 249(14%) | 107(9%) |
| 30–39 years | 125(7%) | 90(8%) |
| 40–49 years | 31(2%) | 63(5%) |
| 50–59 years |  | 68(6%) |
| 60–69 years |  | 95(8%) |
| 70–79 years |  | 47(4%) |
| Spanning multiple ages | 178(10%) | 229(20%) |
| Unidentified | 162(9%) | 115(10%) |
| Sum | 1718 | 1163 |
